# ‘Gushing Out Blood’: Defloration and Menstruation in* Memoirs of a Woman of Pleasure*

**DOI:** 10.1007/s10912-016-9426-0

**Published:** 2016-12-26

**Authors:** Sara Read

**Affiliations:** Loughborough University, Loughborough, UK

**Keywords:** Defloration, Menstruation, Hymenal bleeding, Sex, Medicine, Eighteenth century, Cleland

## Abstract

John Cleland’s 1740s pornographic novel, *Memoirs of a Woman of Pleasure* repeatedly depicts and eroticises the act of defloration. As such it is a revealing illustration of what Ivan Bloch termed the ‘defloration mania’ of the eighteenth century. This article maps narrative events on to contemporary medical depictions of first intercourse to show the ways that the theories and ideas presented in medical and pseudo-medical texts transferred into erotic fiction and demonstrates how in some instances the bloody defloration scenes can be read as being sex during menstruation, an act which was culturally forbidden at this time.

John Cleland’s 1740s novel, *Memoirs of a Woman of Pleasure*, often known simply as *Fanny Hill* after the name of its protagonist, has been described by Tassie Gwilliam as ‘the most famous example of the explosion in erotic and pornographic literature beginning in the seventeenth century’ (1996, 540). As Antje Schaum Anderson has explained, it ‘is structured by episodes that reiterate and restage the experience of defloration for Fanny and others to meet the continual demand for sexual pleasure among characters and readers of *Memoirs* alike’ (1995, 117). This means that the novel can be seen as primarily arranged around this thematic trope rather than a range of sexual activities, even though these also take place. As such it is a classic fictional illustration of what Ivan Bloch in 1938 identified as the ‘defloration mania’ of the eighteenth century (1996). In this craze, wealthy men could, and did, procure virgins for sex for a large fee in bawdy houses (Peakman 2003, 164). This novel is only one example of erotic literature from many which narrate this craze, but in doing so it is exceptional as it describes in detail numerous deflorations, including Fanny Hill’s own several times over and others in lurid, bloody terms. In doing so, it reveals contemporary attitudes to defloration and female bleeding in ways which are not narrated in virtually any other context outside the scope of medical textbooks and guides to reproduction.

Medical treatises produced for both the general public and for practitioners were published in increasing numbers from the second half of the seventeenth century. As Roy Porter has explained: ‘in time a trickle became a flood. There were many such works and they came in various forms, including books for women, books of “secrets”, midwifery manuals, and assorted erotica’ (1995, 35). These texts almost universally state that bleeding is the only true signification of virginity upon first intercourse, even if its absence does not disprove virginity. It is unsurprising, then, that a novel such as *Memoirs of a Woman of Pleasure*, which contains graphic depictions of sexual acts, should reflect the sorts of information offered in the many popular medical treatises, given their wide general readership.

*Memoirs of a Woman of Pleasure* is thought to have been drafted around 1730 in Bombay where Cleland had arrived in 1728 as a teenage soldier in the service of the East India Company (Gladfelder 2012, 18). This makes it approximately contemporaneous with a number of medical texts such as John Maubray’s *The Female Physician Containing all the Diseases Incident to that Sex in Virgins, Wives and Widows* (1724), and the anonymous *A Rational Account of the Natural Weaknesses of the Female Sex* (1716), as well as texts such as the much republished *The Mysteries of Conjugal Love Reveal’d*, a version of a late seventeenth-century text by the La Rochelle-based French physician, Nicolas Venette, despite the fact that the novel did not appear in print until the late 1740s. Texts like Venette’s bridge a gap between serious medical treatises and populist quack pamphlets, aptly described by Porter as being ‘learned but also popular; it is scientific, yet also ribald; it is earnest, it is playful’ (1994, 136). This particular book appeared in at least thirty-one editions before 1800. There is evidence that young men read these sorts of medical treatises and especially the more sensational texts like *Aristotle’s Masterpiece; or, The Secrets of Generation Displayed in all the Parts Thereof* (continuously in print from 1684 until the early twentieth century, appearing in over forty editions before 1800), as pornography rather than as medical texts (Porter 1994, 136). As Mary Fissell relates, John Cannon in his memoirs recalled reading his mother’s copy of *Aristotle’s Masterpiece* as an adolescent in the early eighteenth century in order to find out what women looked like under their clothes (Fissell 2003, 67); similar cases have been identified in America too (Chamberlain 2003, 67).

Some years after the publication of *Memoirs of a Woman of Pleasure*, Cleland himself claimed to be widely read in the new genre of medical advice literature. Indeed, in his mid-eighteenth-century exploration of how to live a long, healthy life, *The Institutes of Health*, he wrote that ‘few eminent writers of that profession have escaped me’; Cleland lists writers from the seventeenth and eighteenth century including Herman Boerhaave and John Freind, along with classical authors as examples of his study (1761, vi). Peter Sabor has commented on the verisimilitude of Cleland’s depictions of physical responses in the novel in relation to the ‘physiology of sexual reactions’ (1985, xxiii), perhaps suggesting that Cleland was well read in contemporary medical and pseudo-medical treatises earlier in his life, too. His use of phrases such as the ‘seat of pleasure,’ a well-known medical euphemism for the clitoris to describe Hill’s sexual arousal is indicative of this as well. It is the case, though, that the depictions of sexual activity in *Memoirs of a Woman of Pleasure* take the sorts of responses described in medical treatises to an extreme to enhance their erotic appeal, but this does mean that the text provides a useful medium through which to examine how ‘hymenal bleeding’, the blood lost on first intercourse, was eroticised and even fetishised, in this era as an extension of the cultural concerns about hymenal blood as a marker of virginity. As I have argued elsewhere, the phrase ‘hymenal bleeding’ is a usefully unifying one to refer to the blood lost at defloration, despite the fact that, as will be discussed, many early modern authors assumed that this blood emanated from the veins in the vagina being agitated and not from a torn hymen (Read 2013, 34).

Defloration happens during vaginal intercourse, which is significant, for as Karen Harvey has shown, non-vaginal sex was not a focus of erotic writing in this era. Anal sex, for example, was ‘unequivocally damned as preposterous and dangerous’ (Harvey 2008, 121). Sarah Toulalan further contends that ‘sexual pleasure was understood as not complete pleasure if it did not have the possibility of conception’ (Toulalan 2007, 64). This article seeks to develop this body of work in two key ways: by mapping the events in the novel onto medical depictions of defloration to show the ways that the theories and ideas presented in medical and pseudo-medical texts transferred into erotic fiction; and secondly, to demonstrate how the blood of defloration was often explained physiologically as being analogous to menstrual blood (Read 2013, 123). Thus, some of the excessive vaginal bleeding in *Memoirs of a Woman of Pleasure* can in fact be explained as menstrual in line with contemporary medical thinking.

A paradigmatic early modern description of the anatomy of the vagina appeared in *Mikrokosmographia: Or a Description of the Body of Man* by Helkiah Crooke in the early seventeenth century: ‘there are two veines which disperse their branches through the wombe, some of which are carried to the inward cavity thereof by which the infant is nourished, others run to the outward part of the wombe even unto the necke and the lap it selfe’ (1615, 315). The neck of the womb in this context is the vagina, rather than the cervix; in the Restoration period, the anonymous author of *Aristotle’s Masterpiece* claimed that the ‘secret’ places in women are called ‘the Neck of the Womb’ by the vulgar (Anon. 1684, 93). Sometimes to prevent confusion, the cervix was called the ‘lesser neck’ with the vagina being the greater neck. This venous understanding of female anatomy is further exemplified in the mid-seventeenth century by the author of *The Compleat Midwives Practice,* which stated that the reason that there are so many veins in the vagina is that ‘the flowers [menstrual periods] must not onely come out of the womb, but out of the neck of the womb also’ (Anon., 1656). These anatomical understandings held into the eighteenth century when John Freind, author of the first monograph on menstruation, described how veins into the uterus and vagina are ‘variously inserted and disseminated, and are “almost infinite in number” but are greater in number in the uterus itself’ (1729, 23–24). Freind went on to explain unambiguously that ‘the Menses therefore flow sometimes from the one, and sometimes from the other, yet very often from them both.’ He expanded upon this to say that menstrual bleeding from the vaginal vessels was more prevalent in virgins than in women who have had children. Restoration physician Thomas Gibson, paraphrasing Soranus of Ephesus, explained that in the course of defloration, these vessels are ruptured and can then bleed profusely:

In Virgins its [the vagina’s] duct is so straight, that at their first congress with a Man they have commonly more pain than pleasure through the extension of it by the *Penis*, whereby some small Vessels break, out of which Bloud issues as out of a slain Victim. (1682, 154)

Interestingly, Hill describes herself as being dressed in ‘gay attire’ while being decked out as the ‘victim for sacrifice’ shortly after her arrival at the brothel (Cleland 1985, 15). Gibson’s last remark clarifies the view that this blood was seen as a form of menstrual bleeding since as far back as the Hippocratic corpus healthy menstrual blood was routinely described as looking like the blood of a sacrificial victim. This connection is why intercourse, for example, was thought to be an unbeatable cure for the virgin’s disease, greensickness, a disease characterised by a lack of menstruation in young women. Whereas nowadays menstrual discharge is understood to be the loss of the lining of the womb, under the humoral system this loss was thought to be an excess of blood which needed to be voided sometimes along with corrupt humours.

So, while the occasion of a woman’s first sexual experience is a time which is often associated with some blood loss, the explanations above show that this loss was, in some readings, thought to be the same blood as menstruation, which itself was imbued with layers of significance in early modern England. This means that the eroticisation of bloody defloration directly engages with the menstrual prohibition which saw the Bible (Leviticus 20.18), medical texts, and social conventions consistently forbidding sex while a woman was bleeding: as medical author John Marten asserted, this was the ‘most forbidden season’ in which to engage in sexual activity (1706, 23). Cultural and religious norms meant, as Patricia Crawford has shown, an abstinence of 1 week was normally advised during the menstrual period (Crawford 1994, 89). The Bible not only forbids intercourse both during menstruation and during the time of post-partum bleeding, but also orders that men and women live separately at this time (Crawford 1981, 62). Medical and cultural texts concurred with the idea of abstinence, which was sometimes termed separation, during menstruation. Even mainstream medical writers such as midwife Jane Sharp writing in her 1671 midwifery guide stated as fact that it was dishonourable for a woman to allow her husband to have sex with her during her period because ‘physicians know that those Children that are begotten during the time of separation will be Leprous, and troubled with an incurable Itch and Scabs as long as they live’ (1999, 45). John Marten also asserted that any resultant conception would produce ‘Weakly or Distemper’d’ children who would be marked out by their red hair (1706, 23). In a later edition of the same treatise, he noted that if only couples would abstain from sex during menstruation they would avoid ‘defiled Conceptions’ and in particular would eradicate small pox and the measles, both of which he put down to the ‘Menstrous Impurities of the Mothers blood’ (1708, 27–28). However, the risk was not just to conception. James Drake’s *Anthropologia Nova; or, A New System of Anatomy*, following the often reproduced myths about the poisonous nature of menstrual blood, explained that, ‘the Malignancy of them [menstrual periods] is so great, that they Excoriate [pull the skin off] the Parts of Men by the Meer contact’ (1707, 322). Only men unable to control themselves would reduce themselves to this act, Marten wrote, claiming that it was only done by those men who were of so ‘hot and ungovernable a Temper as not to spare their wives’ (1706, 23). *Aristotle’s Masterpiece* similarly described how ‘Sailors or Mariners’ who had long been absent from home were renowned for fathering ‘rude and deformed’ children because of their habit of ‘run[ning] upon their Wives without any due regard to their menstrual courses’(Anon. 1684, 49). Despite this cultural approbation, however, in *Memoirs of a Woman of Pleasure*, bloody intercourse is celebrated.

Early modern explanations for the origins of blood loss on defloration meant that once the vaginal veins were opened during first coitus they could be sufficiently agitated to discharge a full menstrual period (Rivière 1655, 403). However, this is not to say that early modern medical texts discounted the idea of a hymen which could tear and bleed. Descriptions of the hymen appear in most medical treatises although as Jane Sharp commented, ‘some do absolutely deny, that there is any such *Membrane*’ (1999, 201). What is clear, however, is that the volume of blood described in pseudo-medical texts, erotic literature and even some serious medical treatises is not commensurate with a torn hymen alone; indeed medical books would relate instances when a woman had died of blood loss following defloration. A translation of respected physician Isband van Diemerbröeck’s *The Anatomy of Human Bodies* by William Salmon in 1694 reported the case of a girl who died on her wedding night from the blood she lost at defloration (Read 2013, 127). It is entirely in keeping with this cultural view that Fanny Hill’s own defloration should lead to her thighs being covered in ‘a stream of blood that flow’d from the wounded torn passage’ (41). Indeed, all the defloration scenes in *Memoirs of a Woman of Pleasure* are shown to be implausibly bloody, which fits with the medical understandings outlined above.

In *Memoirs of a Woman of Pleasure*, defloration is described in various ways including with the protagonist’s own defloration and later the selling of her faked virginity, as well as a number of other characters’ reports of their deflorations. The theme of defloration in eighteenth-century erotic fiction has been discussed critically in terms of the dynastic signification of hymenal blood and its subsequent eroticisation and value as a commodity, and even how it serves to disrupt ‘the traditional linear sexual initiation plot’ (Anderson 1995, 108–138; Gwilliam 1996, 525; Peakman 2003, 165–66). This work has shown that in eighteenth-century society at a basic level, the appearance of blood ‘proved’ a woman’s virginity and thus confirmed that any male line would continue. Julie Peakman has further shown how this was one of the factors which led to hymenal blood becoming an erotic signifier, linked to ‘themes of family honour and masculine territorial rights’ (2003, 165–66). As Joseph Pappa has put it: ‘primogeniture was at stake’, since if women were not virgins on marriage then the family line, the ‘family honour and masculine territorial rights’ of both families was in doubt (2011, 43). An additional and significant factor in its erotic appeal was that the blood of defloration was in reality an elusive sight, often not even present in a real defloration, or as eighteenth-century medical author John Marten expressed it, ‘so much talk’d of, but so seldom seen’ (Read 2013, 143–44; Marten 1735, 24).

So, part of what gave rise to an erotic interest in sex with virgins and the blood associated with this activity was the need for proof from the man’s point of view that he had received what he paid a premium for. One sinister reason for this was undoubtedly the belief that sex with a virgin could cure sexually transmitted diseases, which as Julie Peakman has shown, was a widely-held view in the era (2013, 306). Such attempts at cures were grasped at in this society because diseases such as syphilis and gonorrhoea were rife (Merions 1997, 5). Medical treatises offered cures such as quicksilver (mercury), ointments, medicinal cordials, sweating and rubbing the body, and if these texts mentioned the virgin-cure at all, they advised against it (for example, Boerhaave 1729); Marten even asserted that a patient he knew who had tried this method had ‘render’d his condition worse’ (1706, 23). However, for all such denunciation, as Roy Porter has discussed, the belief was so ‘ubiquitous’ that at the end of the century ‘the medical populariser William Buchan ‘felt compelled to denounced the belief in print saying that the only way to scotch such ‘absurd opinions’ was through popular education’ (1994, 23). Indeed, this theory was so widely believed that Hanne Blank has found that ‘in eighteenth-century London, approximately one in every five capital rape cases on the books involved a victim under the age of ten’ with the ‘virgin cure myth’ given as an explanation in many of these trials (2007, 64). More than this, though, uninfected men sought virgins for sex in order to ensure that they would remain disease free (Gwilliam 1996, 540).

In terms of the narrative arc, as well as thematically, *Memoirs of a Woman of Pleasure* follows the received wisdom announced in the published medical and pseudo-medical treatises which invariably state that girls achieve puberty around the age of fourteen and that it is a gradual process. Fanny Hill, aged fourteen, comes to London to seek a new life upon the death of her parents recording that she ‘was now entering my fifteenth year’, and so in ‘the flower of youth’ (2); although in a narrative slip, a few pages later Hill says that she ‘was barely turned of fifteen’ (14). This means that she was the age that medical texts described as when young women’s thoughts turn to love and sex. *Aristotle’s Masterpiece*, drawing on the work of sixteenth-century physician Levinus Lemnius, explained the connection between the physical signs of puberty and sexual desire:

The propension and inclination of Maids to Marriage, is to be discovered by many Symptoms, as, when Nature fringes the obscure parts, and their Terms flow at the time appointed, which is usually in the Fourteenth or Fifteenth Year of their Age, when the Seed increaseth in some sooner, and in others later, according to their Habits or Constitutions: And the Blood, which is no longer taken to augment their Bodies, abounding, incites their Minds to Venery. (Anon. 1684, 5–6)

The novel, then, shows that Hill is pubescent at the start and her comment that ‘my bosom was finely rais’d, and one might discern rather the promise, than the actual growth, of the round firm breasts, that in a little time made that promise good’, suggests that she is on the point of menarche (14). This sort of sentiment is described in Drake’s early eighteenth-century medical guide *Anthropologia Nova* which states:

About the time of Puberty or Eruption of the *Menses* the Breasts begin to swell, and grow prominent probably from a greater Afflux of Humours at that time, which not only fill the Vessels, but dilate the Substance of them; which opinion is confirm’d by their Shrinking when Age renders them unfit for Procreation, and their *Menses* desert them. (1707, 354)

Thus, Hill is depicted as being at the point where medical treatises contend that girls of her age would have sexual thoughts as she was on the brink of starting her menstrual periods.

It is in this context that the nosebleed Hill experiences is significant. Girls who had not yet begun to menstruate because the passages of their veins were thought to be too narrow might be expected to experience either the symptoms of greensickness—the virgin’s disease–or conversely to experience vicarious menstruation where the blood made egress by means other than vaginally. Helen King has explained that ‘[t]o understand what is happening here in Hippocratic terms we need to remember that menstrual blood was thought to be the excess from the diet. Outside acute fevers, nosebleeds in women were seen simply as menstruation by a longer and less orthodox route’ (1998, 51). The idea of vicarious menstruation was widely accepted in early modernity and referred to in official diagnoses and also in legal trials, for example (McClive, 2002). In Hippocratic teaching, a nosebleed in a young woman such as Fanny Hill would be considered ‘particularly significant—and thus worth noting in a case history—because it should show that the dangerous areas of the body have been traversed [by the menstrual blood], and hence that a certain point of internal physical maturity has been reached’ (King 1998, 73). There was an assumed danger that the blood which was unable to escape the narrow vessels of an immature woman might become lodged in her heart or other internal organs. The cure for both greensickness and vicarious menstruation was the forcible opening of the passages of a woman’s body by excitation in sexual intercourse. This would bring on a menstrual period, and the sufferer would be considered cured. The Hippocratic text *On Generation* states that sexual activity might bring on the menses not just by widening the vessels of the vagina, but that ‘intercourse by heating the blood and rendering it more fluid gives an easier passage to the menses’ (Lonie 1981, 3).

In the account of the first attempt at Fanny Hill’s defloration, an elderly man referred to by Hill as an ‘old goat’ and a ‘monster’, pays the bawd Mrs. Brown for this opportunity (17 and 21). The man suffers from premature ejaculation in his eagerness to achieve his goal but soon recovers himself and makes a second attempt. Much tussling is described as Hill seeks to extricate herself from his groping, and when she calls a maid to come to her assistance she is seized with a massive nosebleed: ‘my hair all dishevell’d, my nose gushing out blood, which did not a little tragedize the scene’ (20). At the sight of this blood, the maid assumes ‘that matters had gone greater lengths than they really had, and that the courtesy of the house had been actually consummated upon me’ (20). Believing that intercourse has taken place, the maid thus advises the man to leave the room so that Hill can compose herself. The blood at the most literal level can be taken as seen by the maid as referent to the hymenal blood that Hill would have lost had the rape proceeded: the nosebleed both substitutes and parodies a defloration (Gwilliam 1996, 532). However, it is also a metaphor for menstrual blood which, as has been discussed, was often thought to begin flowing upon first coition. This scene works at multiple levels to show that the young woman is now sexually mature. However, in order to achieve this, Cleland offers a misogynistic inversion of the notion that thoughts of love and romance are mediators to start a woman’s menses. Although it cannot be the case here that that sexual intercourse has brought on this bleeding, Hill is shown to be a sexual entity, having experienced a physiological response and pseudo-menarche. It is particularly interesting that the novel should do this given that it ignores other social conventions such as avoiding sexual intercourse during a woman’s menses but does avoid breaching the convention that stated that sex before menarche was prohibited (Laslett 1977, 217). The attempted defloration scene, then, serves to bring Hill to sexual maturity and thus render her sexually available. The scene also works at another level, for as Howard Bloch has suggested, ‘according to the Patristic totalizing scheme of desire, there can be no difference between the state of desiring and of being desired, a virgin is a woman who has never been desired by a man’ (Gwilliam 1996, 523). Richard Allestree’s sermon book, published in the same year as *Aristotle’s Masterpiece* makes this connection explicit: ‘Every gross impurity deflowers the soul, and when it sins it plays the strumpet’ (1684, 96). In his earlier text *The Ladies Calling*, Allestree described the connection he saw between impure thoughts representing a defloration of the mind and total ruin: ‘between the state of immaculat Virginity & arrant prostitution there are many intermedial steps, and she that makes any of them, is so far departed from her first integrity’ (1673, 148), thereby insinuating that being desired sexually or having sexual thoughts was the first step to prostitution. The quasi-menstrual nosebleed in the novel acts both as a portent for Hill’s imminent defloration and serves as a trope for her menarche. Thus the nosebleed scene operates to flag to a reader that by being desired, Hill is no longer an innocent virgin of any social worth and sexually mature; therefore, there is no reason she should not become a prostitute.

That the theme of sexual activity stimulating the menses is indeed what is being alluded to in the nosebleed scene is confirmed when this theme is replayed in what Hill terms her second defloration. In a development of the narrative, Hill is now a kept mistress who seeks revenge on her lover for sleeping with her maid. In a tit-for-tat move, she decides to seduce Mr H.’s servant, Will, a country boy recently brought into Mr H.’s employ to run errands between the lovers. Hill discovers that the nineteen-year-old is very well endowed and that this subsequently makes sex difficult, and after a closely narrated act she is left bleeding:

the widen’d wounded passage refunded a stream of pearly liquids, which flowed down my thighs, mixed with streaks of blood, marks of the ravage of that monstrous machine of his, which had now triumph’d over a kind of second maiden-head. I stole, however, my handkerchief to those parts, and wip’d them dry as I could. (76)

Again, this scene deploys an inversion similar to the pseudo-defloration described above, for this time it is the man who is losing his virginity, but for the sake of the titillation of the ritual bleeding of the female, it is Hill herself who suffers a second, bloody defloration. *The Mysteries of Conjugal Love Reveal’d* described the medical theory of how this type of sex could cause bleeding, providing further evidence that Cleland was very familiar with the pseudo-medical texts of the day. This text explains that it is unlikely that the wife of a man with an excessively long penis will conceive, for

the very approach […] puts her into a cruel torment. Indeed, the pain she suffers in being touch’d by it makes her lose her Senses, and quite stupefies her, the Man tearing her *Nymphae* [labia] murdering her *Caruncles* [membranes], splitting the passage, forcing down to the very bottom of the Womb. For which Action ensues a great effusion of Blood, looseness, and other inconveniences that she is exposed to, after having been caress’d in such a manner. (Anon. 1707, 35)

This text goes so far as to suggest that a woman could go to court to argue assault if her husband has so large a penis that it inflicted damage on this scale, but goes on to offer a solution through the manufacturing of a device which stops the penis from penetrating so deeply. The reader was taught to cut a hole in a cork, one or two inches in length as required, and cover it in cotton wadding and linen to which she should attach a string to tie the device around the man’s legs having first placed the penis through the middle of the cork. This would serve to limit the depth of penetration, and the author advised the prudent housewife to make two of these so that she was never caught without one.

In common with the menarche scene, the blood lost at this defloration also proves to be an occasion of menstrual bleeding. Mr H. happens to visit Fanny shortly after her encounter with the servant. Fanny is saved from having to have sex with him—and thus having her recent sexual activity discovered—by her menstrual period, started by the ‘defloration’ in the manner which physician Lazare Rivière had described when he explained that greensick women often ‘have their Terms [menstrual period] the first night after marriage, and that others who are in good health have them before their accustomed time’ (1655, 403). The idea of sexual intercourse causing a menstrual period is one which was later described in an anecdote in the eighteenth-century memoir of William Hickey, born around the time of the publication of *Memoirs of a Woman of Pleasure*. In this episode, the fourteen-year-old Hickey used his pocket money to visit a prostitute who had offered to take his ‘maidenhead’. Hickey described how the prostitute began to menstruate during the sex act:

Upon getting out of bed, however, I was dreadfully alarmed at perceiving the tail of my shirt covered in blood, and screamed out. The poor girl seemed to be in a great agitation and distress, which increased my fright; whereupon she eagerly endeavoured to assuage my fears, assuring me that no sort of injury would arise, that what I saw proceeded from a natural cause, though she had not been aware of it coming on. (1975, 23–24)

Just as in *Memoirs of a Woman of Pleasure*, in this passage too, the young man loses his virginity, but it is the woman whose blood loss acts to mark the defloration. After Mr H. arrives, Hill comments that she was unwilling to have sex with him lest her infidelity be revealed: ‘here the woman sav’d me: I pretended a violent disorder of my head, and a feverish heat, that indispos’d me too much to receive his embraces. He gave in to this and very good naturedly desisted’ (78). Although presented as a ruse—albeit one which would not strike the eighteenth-century reader as implausible—that Hill’s menstrual period did start during sex with Will is confirmed in a further encounter with the servant a month later during her next menstrual period. Hill is surprised by a visit from a suspicious Mr H. ‘[a]bout a month after our first intercourse, one fatal morning (the season Mr H. rarely or never visited me in)’ (84). This coded language which refers at the literal level to the morning, when Mr H. rarely visited his mistress, I would argue confirms that Mr H. would not normally visit his mistress for sex during her menstrual period, making Hill feel safe to indulge in her affair. The ‘season’ in this sense, of course, is the ‘forbidden season’ described by John Marten. The timeline of the narrative confirms the early modern paradigm that the sex with Will had in fact precipitated a menstrual period and the next one followed a month later in the new pattern. This event further eroticises the deviant nature of the affair with Will, if not with Mr H., by suggesting that she would continue to be sexually active with the servant at this time.

Having sex during menstruation with Will the servant also points to Hill’s lack of inhibition: she adheres to none of society’s conventions. Indeed, after being reunited with her lover Charles, Hill holds his erect penis ‘feeling the stiff stake that had been adorn’d with the trophies of my despoil’d virginity’, thus celebrating how it had once been adorned with her blood, shed flamboyantly upon her actual defloration with him (182). Similarly, paying customers are shown to enjoy this unorthodox bloody sex, too. When Hill sells a fake maidenhead to Mr. Norbert and he sees her thighs, shift, and sheets ‘stain’d with what he readily took for virgin gore’ in the morning, far from being repulsed or wary, he is aroused a further time: ‘nothing could equal his joy and exultation’ (136–37). Norbert persuades Hill to let him have further intercourse while he was ‘triumphant and like a cock clapping his wings over a downtrodden mistress’. This shows the power relation played out as the maidenhead-hunter believed he has made another conquest. This celebration and eroticisation of hymenal and menstrual bleeding is very much set against a context in which ‘a seventeenth-century view of menstruation as polluting persisted’ (Harvey 2008, 119). Indeed, John Toland’s eighteenth-century biography of the scholar and Christian martyr Hypatia of Alexandria (d. 415 BCE) relates an episode which shows this blood was not just shunned because of its potential to pollute but also because it was distasteful. Toland repeated the story that in order to dissuade a persistent but unwanted lover, Hypatia showed the man her menstrual blood to repulse him. He described how ‘at that time she happen’d to be under an indisposition ordinary to her sex. She took a handkerchef of which she had been making some use on that occasion, and throwing it in his face, said ‘*This is what you love, young fool, and not anything that’s beautiful*’ (1720, 123). The sight of her menstrual blood was enough to extinguish the young man’s ardour, and so effective was it thought to be that it was given in other medical texts as a cure for lovesickness (Dawson 2005, 465). That claims about the dire effects of menstrual blood on the penis were being produced in literature at this time means that contact with hymenal and menstrual blood must have been seen as a risk-prone activity, further enhancing its erotic potential as a daring act.

To produce the bloody display in the faked defloration scenario, bawd Mrs. Cole shows Hill a secret compartment on the bed which houses a tumbler of a pseudo-bloody fluid and a pre-soaked sponge that she was to anoint herself, her shift, and bed clothes to make it appear as if she had indeed been a virgin to the man who has paid a premium for her maidenhead. Various methods for faking a defloration were discussed in the medical and pseudo-medical texts. These tricks and devices have been discussed by critics in relation to the novel (Boucé 1982, 32; Bernard Yeazell 1991, 109), but the con used by Hill had to be executed after sex when the client is asleep, and the nature of the bloody fluid on the sponge is not disclosed in the novel. John Marten suggested that many women were duplicitous and dishonest and that these sorts of deceptions were well-known, not only with the use of astringents to make the vagina feel tighter and more virginal but also in the more inventive use of props such as those which Hill utilises. Marten suggested that some women ‘when they come to Brides, the better to deceive their Husbands, have either a little blooded their Shifts before-hand, or placed a little Fish bladder of Blood so as to be broke in the Encounter’ or else, Marten suggested, they did indecorously resort to timing their marriages to coincide with their menses (1706, 177). Thomas Gibson agreed that women who married while menstruating could be thought guilty of attempting to trick their husbands because of there being little resistance to penetration but stated that in fact ‘she’s [normally] unblameable saving for her imprudence to marry at that season’ (1682, 155). However, the author of *The Mysteries of Conjugal Love Reveal’d* mused that:

Might it not be allowable for a Woman, who has past some years of her Life in unlawful Pleasures, to secure her Husband’s good Opinion on the Wedding Night by taking up some Blood (which she treasured up before) and putting it into the Privities? May it not be allowable, I say, for the Preservation of Peace in her Family, to take all the pains imaginable to be thought a discreet Woman by her Husband? (Anon.1707, 73)

This appeal contradicts the text’s earlier claim that men ‘do not trouble their Heads about some few drops of Blood that are shed in the first Nuptial Caresses’. Key to the scene in *Memoirs of a Woman of Pleasure* is that there needed to be an ostentatious display of blood to satisfy Norbert’s desires, but this concurrently raised his suspicions that he had been duped. As an experienced ‘maiden-hunter’, Mr. Norbert then examines Hill’s body for signs that she was deceiving him. The sorts of self-contradictory views Norbert embodies, that there should be blood to demonstrate defloration and that copious blood is arousing but yet might suggest he has been conned, as indeed he has, show the complicated symbolism of this blood. Concurrently, it was accepted that intercourse could agitate the vessels of the vagina and provoke a menstrual period leading to larger blood loss, which clouded the significance which could be assigned to the bleeding still further. This explanation highlights again how notions of sex during menses were situated in a strong cultural linkage with hymenal bleeding. Whereas some blood would ‘prove’ virginity on consummation of a marriage, or defloration, too much blood lubricating the vagina could arouse suspicions that the woman was trying to trick her husband/client.

All medical texts argued that hymenal bleeding ‘shed in the first Nuptial Caresses’ was an absolute proof of defloration; however, most texts also stressed that a lack of bleeding did not signify that a woman was not a virgin. Cleland rather disingenuously signals this convention when Hill remarks that ‘as to the usual bloody symptoms of defloration, which, if not always, are generally attendant on it’ (132). This points to the event having a greater significance as a cultural construct than as a real physiological event.

*Memoirs of a Woman of Pleasure* is, as I have shown, a text which is explicit in its linking hymenal bleeding, ‘virgin-flower’ (40), to menstruation, known most commonly in early modernity as ‘the flowers’ (Read 2013, 34), in line with early modern cultural assumptions about where this blood comes from and why it appears. Menstrual blood was also a signifier of fertility; as Karen Harvey has written, menstruation was considered ‘dangerous yet cherished’ in society generally, but in erotic literature adds to the hint of danger with the knowledge that a pregnancy might occur (2008, 118). Sex involving hymenal/menstrual blood shows that the woman is both fertile but not yet pregnant which adds to the eroticism of the act. Menstruation and vaginal bleeding were something women ordinarily tried to be discreet about, even in an era in which personal bodily privacy was difficult to achieve. Physician James Drake’s anatomy guide described how an unexpected menstrual period staining her garments was an ‘indecent Surprize’, which occurrence would put ‘otherwise modest and careful Women [in] to such Confusions and Shifts’ (1707, 325). Later in the eighteenth century, the physician Malcolm Flemyng also commented that some women have no symptoms to alert them to the start of a period, so that they ‘scarce have warning enough to provide for decency’ (1759, 351). It is natural, then, that this should become eroticised as secret or forbidden. That virtually all the sexual contact in this novel involves blood, including the scenes of flagellation, further suggests an erotic link to a blood taboo (Peakman 2003, 156). The biblical and medical imputations about the dire consequences of contact with menstrual blood add an erotic thrill to this imagery. Furthermore, sex with a virgin was thought to be a good way to remain free from sexually transmitted diseases, for which gentlemen were prepared to pay a handsome premium, but yet sex while bleeding was thought capable of causing diseases of the penis, then this might be an occasion for the thrill of a controlled risk: examples of men contracting penile cancer and leprosy from sex were rare, while venereal disease was almost epidemic. All of the above shows what a complex signifier bloody sex was.

Medical texts in their continuous arguments about the nature and source of this blood demonstrate further assumptions about female physiology as being problematic and impossible to properly identify. But as Julie Peakman has argued, ‘[t]he interest in defloration was dependent, in part, on the excitement aroused by blood’ (2003, 160). The range of explanations offered for the significance of this blood: power, primogeniture, fertility, taboo, and even healing (of pox-ridden men and greensick women) are diffuse but all connected to eighteenth-century expectations and assumptions about the female body. Hymenal blood was meant to signify that a woman had lost her virginity, but as Tassie Gwilliam has stated, *Memoirs of a Woman of Pleasure* is a novel which compromises the notion that defloration is an absolute transformation, changing a maid to a woman (1996, 351). Sex with a prostitute is not about transforming a maid into a wife, of course, but an elaborate and conscious fantasy version of the traditional wedding night act (‘a copy of a wedding’ as Hill describes it [131]). The theatrical defloration rituals, for which maiden-head hunters paid a premium, show how far bloody sex had become an erotic symbol. The eroticisation of prohibited vaginal blood works to complicate notions that intercourse during vaginal bleeding was both dangerous and indecorous. This was perhaps part of the appeal of the novel, providing erotic, voyeuristic depictions of acts which men found thrilling as a fantasy, but which they might not care or have occasion to do in life.

At the end of the novel in a further narrative twist, instead of ending up ruined by her lifestyle, Fanny Hill is happily married to her first love, Charles, meaning their initial lovemaking was prophetic of the novel’s dénouement and the transformation of Hill into a wife. Antje Schaum Anderson has argued that this means, in narratological terms, that in the end the novelty of the plot is defeated by ‘a male, linear narrative into the hegemonic, patriarchal heterosexuality’ (1995, 120). Importantly, however, it is conforming to the stereotype put forward in the anonymously published Venette’s pseudo-medical text that ‘there are few Women but what love those desperately that obtain the first Favours from them. They are ty’d to their first Lover’ (Anon. 1707, 206). The ending then demonstrates the overlap between the erotic and sensationalist medical texts and erotic fiction.
